# Postoperative adhesions: are we close to finding a solution?

**DOI:** 10.52054/FVVO.15.4.112

**Published:** 2023-12-13

**Authors:** P.R. Koninckx, E Saridogan, V Gomel

**Affiliations:** Prof emeritus Obstetrics and Gynecology KULeuven, Leuven, Belgium, the University of Oxford, Oxford, UK, University Cattolica, Rome, Italy and Moscow State University, Moscow, Russia; Elizabeth Garrett Anderson Institute for Women’s Health, University College London, United Kingdom; Department of Obstetrics and Gynecology, Faculty of Medicine, University of British Columbia, Vancouver, British Columbia, Canada

## Introduction

Postoperative adhesion formation and its prevention remains a challenging clinical problem, as discussed in this issue by the working party of European Society for Gynaecological Endoscopy, Adhesions Research Special Interest Group (Torres-De La Roche et al., 2023). As the authors explain, postoperative adhesions occur in up to 95% of women after surgery, and can cause chronic pelvic pain, infertility, small bowel obstructions, sometimes requiring repeat surgery with more complications. Healthcare costs are substantial, increasing linearly for at least ten years.

Reducing the burden of adhesions must start by prevention since adhesiolysis is poorly effective, possibly because of permanent fibroblast reprogramming. However, without animal models reflecting the complexity and duration of human surgery, we cannot expect to understand the relative importance of the surgeon’s skills, the duration of surgery, the many factors involved and their interaction.

## Adhesion formation is multifactorial and poorly predictable

For most surgeons, adhesion prevention can be summarised as good surgical practice with microsurgical gentle tissue handling and eventually a barrier at the end of surgery. It is not surprising that the awareness of adhesion is low (Torres-De La Roche et al., 2023) since after having done surgery as well as possible, adhesions are perceived as fate. Moreover, the problems of adhesions are diagnosed much later and generally by other physicians. A prediction of the individual risk, e.g. the CLASP system, would be helpful to individualise in which patients antiadhesion agents are needed.

However, we fear that an accurate prediction model could be elusive without an animal model reflecting human surgery which may last for several hours and difficult to validate without an accurate non-invasive diagnosis in humans of the various types of adhesions, from filmy to dense, innervated, or vascularised. In addition, interventions that can be undertaken by the surgeon are limited to general principles, since it is prohibitively complex to investigate the many factors involved that are listed by Torres-De La Roche et al. (2023) and their interaction. The investigation of 2 factors simultaneously in a randomised controlled trial needs a factorial design. Two levels (Y/N) of each factor result in 2*2 = 4 groups, and three levels (no, +,++) already in 3*3=9 groups to be randomised. A trial of 3 or 4 factors and their interactions is no longer realistic (Koninckx et al., 2023).

## During surgery: ‘Gentle tissue handling’

Torres-De La Roche et al. (2023) emphasise the microsurgical principles of gentle tissue handling as pioneered by Victor Gomel in the early 1980s (Gomel and Koninckx, 2016; Gomel, 2019). These recommendations were based on clinical experience and repeat laparoscopies in a Bayesian approach using animal research data and sequential learning in humans. It took us 25 years to understand and validate this concept through research on the pathophysiology and homeostasis of the peritoneal cavity and the function of the mesothelial lining and its repair (Koninckx et al., 2016). The surgeon should understand that adhesions result from the two mechanisms involved: acute inflammation of the entire peritoneal cavity and inflammation of the surgical area. Mesothelial cell reacts within seconds to minimal injury by retraction, breaking the integrity of the mesothelial layer and causing acute inflammation, with exudation of fluid, larger molecules including coagulation factors, recruitment of neutrophils and depression of fibrinolysis. Clinically, clotting and neutrophils seem efficient mechanisms to limit and fight a peritoneal infection. A surgical injury results in local inflammation and a programmed cascade of events, covering a denuded area within hours, removing fibrin and repairing the mesothelial layer. If repair is not completed in 3 days, fibroblasts start to grow, and after five days, angiogenesis starts.

Surgery exposes the peritoneal cavity to CO_2_-inducing mesothelial cell hypoxia or to air with 20% oxygen, causing severe oxidative stress. Both cause acute inflammation, increasing with time and insufflation pressures. Saline toxicity on mesothelial cells, and increased fibrin deposition by desiccation were already known in 1971 (Gomel, 2019). Even delicate grasping damages the mesothelial cell, but stronger grasping causes tissue damage and inflammation, as does surgical trauma.

Therefore, adhesion prevention during surgery needs a combination of factors. The duration of surgery should be short and the insufflation pressure low, ideally below 10 mm Hg, if the pneumoperitoneum is sufficient to permit safe surgery. Desiccation should be avoided, and the gas should not be heated since a lower temperature decreases metabolic activity and the trauma of hypoxia or oxidative stress. The most effective factor is adding 10% of N_2_O (Corona et al., 2017). Saline should be replaced by a more complete liquid, such as Ringers. Mechanical trauma should be limited by minimal grasping with little force. To reduce the duration of the inflammation of the surgical trauma, necrotic tissue and suture material should be minimal with no infection and leaving little blood and fibrin after surgery. Practically, surgical energy should be used judiciously, and sutures should be thin with minimal volume of knots without strangulating tissues. Due to fibrin sticking to the mesothelial cell that is difficult to wash off, the use of abundant irrigation with some heparin is necessary.

The future may bring in new concepts such as adding 10% N_2_O, better and medicated rinsing fluids (Adamyan et al., 2015), understanding the trauma of capillary compression and reperfusion, well-known in transplant surgery, and opium-free anaesthesia (Mulier and Dillemans, 2019). The use of a shot of dexamethasone after surgery will need better understanding; it was used in microsurgery and proven to be effective in animal experiments after conditioning, probably by decreasing fibroblast growth, which is specific for dexamethasone. It is not clear whether anti-inflammatory drugs are helpful.

## At the end of surgery: barriers and flotation agents

Torres-De La Roche et al. (2023) discuss using barriers and flotation agents at the end of surgery to decrease adhesion formation. They are believed to be effective by keeping tissues separated. However, their role in limiting adhesiogenic substances in peritoneal fluid from reaching tissues or by diluting peritoneal fluid is not yet understood. Although the efficacy in decreasing adhesions by 50% is well demonstrated for selective interventions in humans, an improvement of clinical endpoints such as chronic pelvic pain, infertility or reoperation rates has still to be demonstrated.

Most barriers are poly-sugars such as hyaluronic acid, cellulose or starch based. They are viscous by cross-linking or powders rapidly adsoring water to become a gel. Clinicians should know that the resorption can slightly increase CRP, up to mimicking a bowel perforation, and that granulomas can occur if used in large quantities (Krentel et al., 2023).

## Conclusion and discussion

As demonstrated in microsurgery, adhesion-free surgery was achieved by combining all known preventive factors for severe surgery, such as deep endometriosis excision (Koninckx et al., 2013). Practically, this consisted of humidified CO_2_+ 10% N_2_O at 30° C, Ringer’s lactate for rinsing, clean CO_2_ laser excision, thin sutures, strict haemostasis avoiding coagulation of blood in the pelvis, a barrier at the end of surgery and dexamethasone administration.

Besides good surgical practice, it is estimated that preventing mesothelial cell trauma and acute inflammation can be estimated to reduce adhesion formation by 70% to 80%, barriers by 50%, and both together by over 95%.

The surgeon has to decide how to organise adhesion prevention in the individual patient. Not to use heated gas or saline seems solidly established. The addition of 10% N_2_O is not yet readily available. Rinsing is preferable to decrease fibrin deposition, but not rinsing to facilitate retroperitoneal dissection. Gauses were banned by microsurgery but can be useful during extensive laparoscopic surgery. It remains unclear whether barriers or dexamethasone can be given when there is a risk of infection or bowel perforation. We should realise that practical adhesion prevention is a progressive clinical Bayesian process of improvement by the surgical community.

Intrauterine adhesions are not discussed here since their pathophysiology is different. Furthermore, the prevention of recurrences after adhesiolysis and whether adhesions should be excised is not discussed in the absence of solid data.

In conclusion, training and education are important since the duration of surgery and the trauma and suturing vary with the surgeon’s skill. These data also emphasise the importance of clinical adhesion prevention, which is multivariate and often experience-based (Wattiez et al., 2023), with ‘gentle tissue handling’ as a historical example (Gomel, 2019).
